# Hierarchical Statistical Models to Represent and Visualize Survey Evidence for Program Evaluation: iCCM in Malawi

**DOI:** 10.1371/journal.pone.0168778

**Published:** 2016-12-30

**Authors:** Jamie Perin, Ji Soo Kim, Elizabeth Hazel, Lois Park, Rebecca Heidkamp, Scott Zeger

**Affiliations:** 1 Department of International Health, Johns Hopkins University, Baltimore, MD, United States of America; 2 Department of Biostatistics, Johns Hopkins University, Baltimore, MD, United States of America; Kenya Medical Research Institute - Wellcome Trust Research Programme, KENYA

## Abstract

Policy and Program evaluation for maternal, newborn and child health is becoming increasingly complex due to changing contexts. Monitoring and evaluation efforts in this area can take advantage of large nationally representative household surveys such as DHS or MICS that are increasing in size and frequency, however, this analysis presents challenges on several fronts. We propose an approach with hierarchical models for cross-sectional survey data to describe evidence relating to program evaluation, and apply this approach to the recent scale up of iCCM in Malawi. We describe careseeking for children sick with diarrhea, pneumonia, or malaria with empirical Bayes estimates for each district of Malawi at two time points, both for careseeking from any source, and for careseeking only from health surveillance assistants (HSA). We do not find evidence that children in areas with more HSA trained in iCCM are more likely to seek care for pneumonia, diarrhea, or malaria, despite evidence that many indeed are seeking care from HSA. Children in areas with more HSA trained in iCCM are more likely to seek care from a HSA, with 100 additional trained health workers in a district corresponding to a 2% average increase in careseeking from HSA. The hierarchical models presented here provide a flexible set of methods that describe the primary evidence for evaluating iCCM in Malawi and which could be extended to formal causal analyses, and to analysis for other similar evaluations with national survey data.

## Introduction

Rapidly changing implementation contexts add complexity to the evaluation of public health policies and programs. Across many low- and middle-income countries (LMIC), populations are experiencing dramatic epidemiologic transitions including an accelerated decline in child mortality due to infectious disease and undernutrition [1]. These trends are paralleled by changing investment priorities for interventions aimed at improving the health of women and young children [2]. LMIC governments, donors and other stakeholders need to know if and how investments are producing desired results. In practice, however, it is difficult to isolate and systematically assess the impact of policies and intervention strategies implemented in complex environments. Multiple interventions targeting the same outcomes (e.g. mortality, nutritional status) may be implemented in different ways at the same time at national and sub-national levels.

Many LMIC have available data sources related to intervention implementation, coverage and impact that are underutilized for policy and program evaluation applications including nationally-representative surveys (e.g. Demographic and Health Survey (DHS), Multiple-Indicator Cluster Survey (MICS)) and routine service provision (e.g. data compiled through a Health Management Information System (HMIS)). The National Evaluation Platform (NEP) is a new approach to evaluate maternal, newborn, child health, and nutrition policies and programs that aim to support LMIC governments in answering their priority evaluation questions by compiling, analyzing and interpreting these data using methods that support valid causal inference. NEPs are currently being developed and tested by governments in four countries in sub-Saharan Africa, Malawi, Mali, Mozambique, and Tanzania, with technical support from the authors.

This paper is about NEP statistical methods for analyzing survey data to quantify evidence in support of competing hypotheses implied by a national health policy question. Discussion of analyses that incorporate routine service data from HMIS and other sources are left to subsequent research. The objective here is to describe observed associations between measures of policy implementation and their intended impact for program evaluation using common survey sources including DHS and MICS. This description and summary of evidence is a precursor to formal causal or counterfactual interpretation of the evidence.

We present this synthesis of evidence through a case study evaluating the introduction and scale-up of an Integrated Community Case Management (iCCM) for childhood illness intervention by trained community health workers in Malawi. Our core methods including data management and analysis tools in standard software are made available to national stakeholders in all four NEP countries. Although we are motivated primarily by the needs of governments and institutions related to the NEP, we believe the lessons learned will be applicable to other more general program evaluations, especially those at the national level.

### Use Case: Community Case Management of Childhood Illnesses in Malawi

iCCM is a package of diagnostic, initial treatment and referral interventions targeting pneumonia, diarrhea, and malaria delivered by frontline community health workers [3, 4]. The aim of the iCCM strategy is to improve treatment access among populations not reached through health facility-based services. iCCM has been shown in smaller experiments to improve child health and reduce child mortality [5]. However, recent evaluations of nationally scaled-up versions have been mixed [6, 7].

Malawi was an early adopter of iCCM, first in 10 districts in 2009 and then for all 28 districts starting in 2010 [8]. The Malawi Ministry of Health (MOH) has a long-standing cadre of paid community health workers called Health Surveillance Assistants (HSA) posted in communities throughout the country who implement health and sanitation interventions. Under the iCCM program, a portion of these HSAs, primarily those who worked in areas designated as “hard-to-reach” by the MOH in terms of health facility access, were identified, trained and deployed to provide iCCM [9].

### Evaluation questions

The main evaluation question was whether the introduction of iCCM improved access for the treatment of pneumonia, diarrhea and malaria in young children. We asked whether there was improved access to care for these illnesses only from HSAs, and then separately, whether there was improved access to care from any source. The primary evaluation of iCCM in Malawi has recently been conducted [10]. We revisited this evaluation to further investigate the evidence for iCCM in Malawi, and to make recommendations for future evaluations of iCCM and other similar programs.

**Fig 1:**
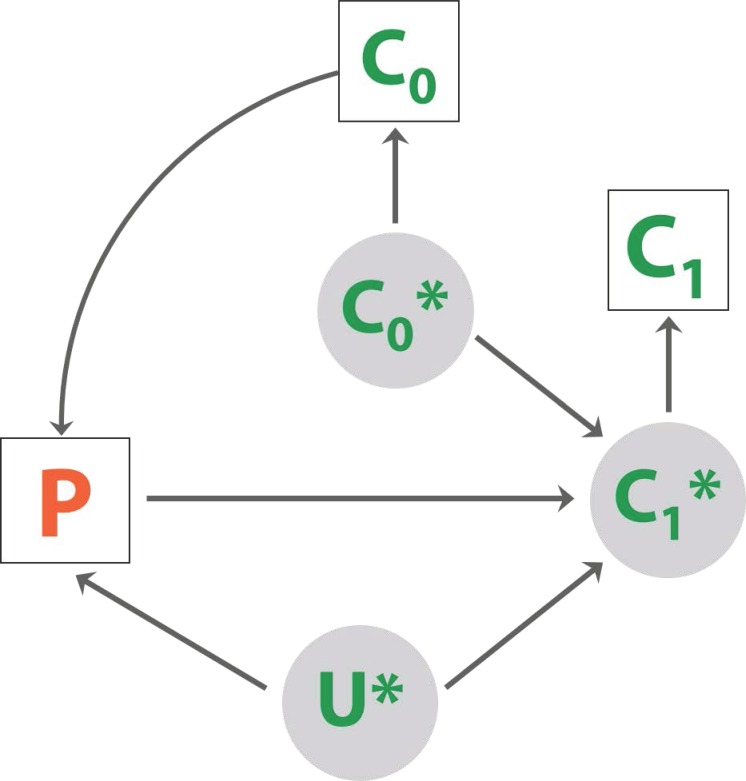
Evaluation framework. Conceptual framework for the evaluation of CCM in Malawi, with careseeking in 2014 as the primary outcome of interest, represented as actual (C_1_*) and measured (C_1_). Baseline (2010) careseeking is represented as actual (C_0_*) and measured (C_0_). We assume that careseeking in 2014 is related to careseeking in 2010 and program implementation of iCCM (P). Program implementation may be determined by baseline careseeking, and potentially by unobserved factor U*.

Fig 1 illustrates the primary question, acknowledging that the program intensity in each district might be influenced by the observed survey data from 2010. In other applications, evaluation may need to account for the non-random allocation of the intervention to the geographic areas. There may also have been important, unmeasured confounding factors U that influenced both the intensity of the program P in a district and the actual coverage attained C*. This possibility and the degree of confounding will be the focus of a planned causal evaluation.

## Methods

### Data sources

We obtained data on careseeking for malaria (fever illness is presumed malaria), suspected pneumonia (acute respiratory infection) and diarrhea by provider type (e.g. HSA or facility) from two large household surveys conducted in 2010 [11] and 2014 [12] among children 2 to 59 months old. In each survey, roughly nineteen thousand child-mother pairs in fourteen thousand households were enumerated in 849 (2010) and 1,139 (2014) primary sampling units. The island district of Likoma was excluded from this analysis because neither survey sampled households from this region. We used the number of iCCM-trained HSAs per district as a measure of the extent of iCCM implementation in each of these 27 districts along with the district population of children under five. These data were obtained from a 2013 telephone census of all iCCM trained-HSAs in country conducted by the National Statistics Office [8].

### Generalized linear mixed models of survey data

A platform for describing and visualizing the evidence relevant to assessing the impact of health programs at the national scale needs to account for the diverse levels at which programs are implemented and where program impacts occur in space and time. We focused here on the motivating iCCM evaluation in Malawi, however, generalized linear mixed models (GLMM) are easily tailored to other programs. A detailed treatment of GLMMs is provided by Gelman and Hill [13] and McCulloch and Neuhaus [14]. We described the association between careskeeing by the mother of a sick child (outcome variable) and the intensity of the program implementation, specified by the number of iCCM-trained HSAs posted in each Malawi district in mid 2013 and the district population of children under five. The model assumes that each district in each year has an unobserved effect representing the extent to which the careseeking behavior in a given year was greater or less than the national average. We used a likelihood-based framework to estimate these modeled effects. To account for survey design, we also included a random effect for each primary sampling unit or census enumeration area (EA). Since, to seek care or not is a binary outcome, we used the canonical logistic model.

This model is termed “hierarchical” given that an observed child is nested within family, sampling unit, district, and time. Given these random effects, the probability of careseeking (*μijkm*) for a given child is

$$logit(\mu_{ijkm})=\alpha ’\;W_{ijkm}+\beta_{1}\;I{\left(2014\right)}_{i}+\beta_{2}\;HSA_{j}+\beta_{3}\;Log(U5\;Population_{j})+\gamma HSA_{j}\;I{\left(2014\right)}_{i}+\delta_{ij}+\eta_{ijk}$$ (1)

for child *m* in survey *i*, either 2010 or 2014, district *j* = 1,*…*, 27, and primary sampling unit or enumeration area *k*. **W***ijkm* is a matrix of child level characteristics including age and mother’s education with a vector of coefficients **α,** potential confounders *U* (Fig 1). The year of survey is indicated by *I*(2014)*i*, which is 1 in 2014 and 0 otherwise. The number of HSA in district *j* is represented by *HSAj*, which may have been related to careseeking in 2010 depending on the rollout strategy for where HSA would be located [8]. This relationship can be framed as *C*_*0*_ causing *P* (Fig 1). The population of children in district *j* in 2012 as projected by the 2008 census is represented by *U5 Population*_*j*_, which may have influenced careseeking as a confounder *U* (Fig 1). Random effects were specified by *δ*_*ij*_, for district, and *η*_*ijk*_ for enumeration area.

Program implementation, or *P* (Fig 1), is represented by *HSAj*, and the primary evaluation target is *γ*, the coefficient for the interaction between the number of HSAs posted in district *j* and survey year, *I*(2014)*i*, represented by the relationship between *P* and *C*_*1*_ (Fig 1). This model assumed that rate of careseeking for children of similar age and maternal education varies on a logit scale across districts, enumeration areas and families according to a distribution at each level reasonably approximated by the normal or Gaussian.

We specified the relationship between the probability of seeking care and district under-five population using the natural logarithm of the population (Model A). We assessed model sensitivity to extreme observations by excluding Zomba district, because of its relatively large number of HSA (Model B). We included a model of the response relationship between probability of seeking care and under-five population using a spline function of the logarithm (Model C), and lastly recreated this model while excluding Zomba district (Model D). Additional sensitivity analyses for the functional form of the number of district HSAs are included in an annex.

### Quantifying and visualizing evidence

The primary association of interest is specified in Eq (1) by *γ*, the coefficient for the interaction between the number of district HSAs (or functions thereof) and an indicator for the 2014 cross-sectional survey. This parameter allows that districts with more HSAs had correspondingly greater increases from 2010 in careseeking from HSAs, controlling for child, maternal and household characteristics and district attributes including the estimated 2010 level of careseeking and district population.

We first used Eq (1) to describe the association between program implementation and the careseeking for childhood illnesses from HSA. In addition, we examined the correlation between program and careseeking from all sources, including HSA and health facilities, as in the primary evaluation of iCCM in Malawi [10]. For both these, we conducted sensitivity analysis for the relationship between careseeking and district population of children under five years of age. All analysis was conducted in R [15]. Code to reproduce this analysis with simulated test data is available online at http://www.jhsph.edu/research/centers-and-institutes/institute-for-international-programs/index.html.

## Results

**Fig 2:**
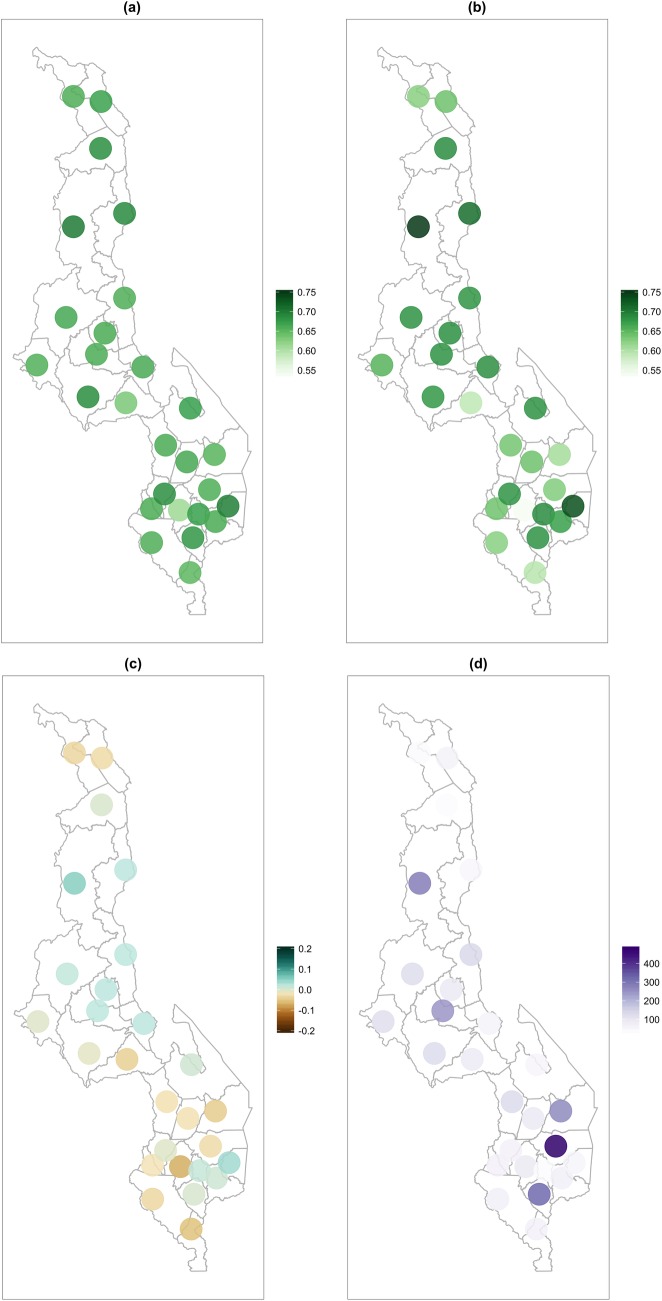
District careseeking from HSA. Estimated careseeking from HSA for children 2–59 months old among those sick with pneumonia, diarrhea, or malaria in the 2010 Demographic and Health Survey (a) and the 2014 Multiple Indicator Cluster Survey (b) and the change from 2010 to 2014 (c) by district, as well as the number of Health Surveillance Assistants active in each district (d).

There were 3,386 deployed iCCM-trained HSAs identified by the census conducted in 2013. The number of HSAs per district ranged from 40 in Rumphi to 497 in Zomba, with a district average of 125. The number of HSA for all districts is shown in a map of Malawi in Fig 2. The average estimated careseeking from HSAs among children with symptoms of diarrhea, malaria, or pneumonia in the 2010 survey was 2.6% compared to 10.4% in 2014. Careseeking from all sources among these same children is 67.6% in 2010 and 68.1% in 2014.

### Careeksing from HSA

We first used household survey data from 2010 and 2014 to estimate the relative odds of seeking care from an HSA for pneumonia, diarrhea, and malaria as a function of the number of HSAs available in each of the 27 Malawi districts, controlling for district population, and the previously described characteristics of the mother and child. The coefficient for the HSA effect in 2014 is controlled for the latent district random effects from 2010, because the model allows for correlation among the district random effects for the two surveys. Because the number of HSAs for Zomba was an extreme outlier, we estimated each model twice, using survey data from all 27 districts, and separately for only 26, excluding Zomba district. Results are shown in Table 1. The change in average careseeking associated with a change in the number of HSA is represented by the term nHSA x I(2014) in Table 1.

**Table 1:** Results for average careseeking from Health Surveillance Assistants (HSA) in Malawi in 2010 (reference) and in 2014, conditional on child age, mother’s education, district population of children under five, and the number of HSA. Models A and C specify the logarithm of district under five population, and models B and D specify a spline of the log of under five population. Models A and B are for all districts in Malawi except Likoma, while Models C and D are for all districts except Likoma and Zomba districts.

	Model A		Model B		Model C		Model D	
	Est	SE	Est	SE	Est	SE	Est	SE
*Fixed Effects*								
Intercept	-4.79	0.23	-4.93	0.38	-4.35	0.23	-4.35	0.40
nHSA x I(2014) (hundreds)	0.22	0.11	0.22	0.11	0.61	0.22	0.59	0.21
nHSA (hundreds)	0.10	0.12	-0.02	0.11	-0.24	0.16	-0.39	0.17
I(2014)	1.45	0.19	1.45	0.19	0.93	0.29	0.98	0.28
Log(U5 Population)	-0.08	0.17			-0.05	0.15		
Spline of Log(U5 Population)								
Term 1			0.99	0.35			1.08	0.38
Term 2			-0.44	0.77			-0.68	0.79
Term 3			-1.24	0.42			-1.09	0.44
*Random Effects*								
EA Variance	1.918		2.058		2.060		2.075	
District Variance	0.126		0.050		0.278		0.194	

Districts with many HSA relative to their population of children 2 to 59 months tended to have larger increases in careseeking from HSA for child illnesses. This association was observed across different assumptions for the relationship between under-five population and careseeking, and was maintained when Zomba district, the area with the most iCCM trained HSAs, was excluded. An additional 100 HSAs in a district was associated with an increase in average careseeking from HSA of 0.27 on the logit scale (Model B in Table 1), approximately 2% on the probability scale.

When Zomba was excluded, this association becomes more extreme, increasing from 0.27 to 0.61, indicating that the estimated increase in careseeking associated with more HSA is not due to the large number of HSA or other conditions in Zomba, and reflect general circumstances related to active HSA on average across Malawi. We selected the best model for the change in careseeking from HSA using Akiake’s Information Criteria including survey data from all districts, which identified Model B.

### Careseeking from all providers

**Fig 3:**
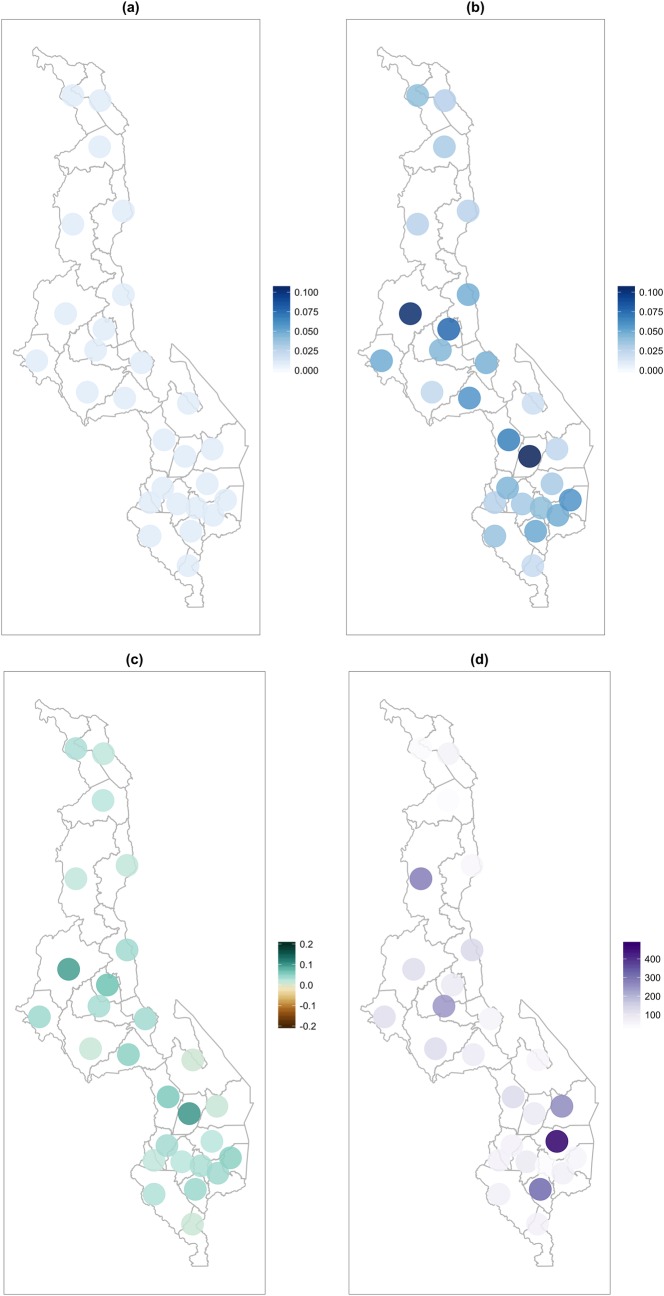
District careseeking from all sources. Estimated careseeking from all sources for children 2–59 months old among those sick with pneumonia, diarrhea, or malaria in the 2010 Demographic and Health Survey (a) and the 2014 Multiple Indicator Cluster Survey (b) by district, and the change from 2010 to 2014 (c), with the number of Health Surveillance Assistants active in each district (d).

We also examined the association between the number of active HSA in a district and the careseeking for childhood illnesses from all sources, including HSA and health facilities using the same methods with hierarchical logistic regression while adjusting for child age, mother’s education, urban or rural area, and population under five. In contrast to careseeking from HSA, there was not an association between the number of HSA and average overall careseeking, either when including all 27 districts for which there was survey data, or when Zomba district was excluded. Results for this hierarchical model are shown in Table 2. The estimated association of an additional 100 district HSA is a slight decrease in careseeking from all sources of -0.01 in the logit scale, a decrease of approximately 0.2% in the probability scale. Estimated careseeking from all sources in 27 districts in Malawi for 2010 and 2014 are shown in Fig 3, along with the number of active HSA in each district.

**Table 2:** Results for average careseeking from any health provider in Malawi in 2010 (reference) and in 2014, conditional on child age, mother’s education, district population of children under five, and the number of HSA. Models A and C specify the logarithm of district under five population, and models B and D specify a spline of the log of under five population. Models A and B are for all districts in Malawi except Likoma, while Models C and D are for all districts except Likoma and Zomba districts.

	Model A		Model B		Model C		Model D	
	Est	SE	Est	SE	Est	SE	Est	SE
*Fixed Effects*								
Intercept	0.70	0.08	1.20	0.16	0.71	0.09	1.19	0.16
nHSA x I(2014) (hundreds)	-0.01	0.05	-0.01	0.05	0.03	0.07	0.03	0.07
nHSA (hundreds)	0.01	0.04	0.04	0.04	0.02	0.06	0.06	0.06
I(2014)	0.00	0.08	0.00	0.08	-0.04	0.10	-0.04	0.10
Log(U5 Population)	-0.30	0.06			-0.31	0.06		
Spline of Log(U5 Population)								
Term 1			-0.73	0.16			-0.77	0.17
Term 2			-1.00	0.32			-1.00	0.32
Term 3			-0.49	0.14			-0.53	0.15
*Random Effects*								
EA Variance	0.27		0.26		0.26		0.26	
District Variance	0.02		0.010		0.02		0.01	
District x I(2014) Variance	0.03		0.040		0.03		0.03	
Correlation	0.24		0.380		0.21		0.29	

## Discussion

We used data from two large cross-sectional and nationally representative surveys to summarize district level rates of careseeking in Malawi for children with diarrhea, pneumonia, or malaria over time from all sources and only from HSA. We used this evidence to compare across areas relating to iCCM program implementation determined by the number of active HSAs in each district. We fit a selection of models to describe careseeking among sick children in Malawi, reporting detail on a representative subset of models. We found that having more HSA relative to the population of children under five was associated with higher average careseeking only from HSA, however, we did not observe an association between careseeking from all sources and the population-adjusted number of district HSA. This finding is in agreement with Amouzou et al. that the evidence was not consistent with a large impact of the iCCM program for careseeking in children aged 2 to 59 months [10].

It is tempting to interpret the observed association in counterfactual terms, that is, indicating what would have happened had iCCM not been implemented. However, the potential for confounding limits what can be concluded about iCCM. To determine how careseeking behavior would have manifested in the absence of iCCM, it is critical to assess the potential for a confounder to influence the estimated association between careseeking and the number of district HSA, labeled U* in our causal diagram (Fig 1). Although a specific confounder was not identified here, there are several candidates, such as the number of health facilities or the quality of the health system, which were described in a recent Service Provision Assessment for Malawi [16], and other potentially unmeasured confounders [14]. We have summarized the evidence related to the implementation of iCCM and the information for making a causal determination, however, the evaluation of iCCM with this evidence is left for further research.

This analysis could be extended in several ways. First, we did not pursue the geographic modelling of random effects, although these methods have been used for cross-sectional survey data [17]. We also did not examine household surveys from other countries, so the generalizability of this approach outside Malawi is still a matter for further research. Both surveys examined here were designed to be representative at the district level, the unit where program implementation was observed and a critical unit in terms of routine or HMIS data [18]. We would like to employ these methods for other potentially sparser surveys, using HMIS data to indicate program implementation. Our research is also interested in using DHS surveys for evaluation that were conducted prior to 2009, when the location of enumeration areas may have been displaced outside their district boundaries [19]. This displacement presents an additional challenge to the low statistical power in many surveys for making inference in subnational areas.

In addition to these planned extensions, there are also limitations in this analysis regarding the interpretation of the main parameters of interest, namely, the estimated association between program implementation and district average careseeking, which are conditional on the circumstances of each district. The parameter estimates of interest are also dependent on the estimated hierarchical effects across districts and their assumed distribution.

For many countries, multiple surveys are available for monitoring and evaluation of health programs. The health information in these surveys represent an extensive resource for analysis and for greater understanding of the outcomes of health programs worldwide, in a time where program evaluation is becoming more complex. We propose statistical methods that are widely used by public health researchers to summarize these survey data, to further understand the evidence relating to iCCM implementation in sub-Saharan Africa, and to promote the understanding of health programs in general.

A formal causal analysis and evaluation of iCCM could proceed from the analysis here in one of several directions. Analysis could control for confounders via statistical manipulations from the field of causal inference [20] for example using an estimate for the probability of treatment or by matching units or areas based on specific attributes. An evaluation could also proceed by identifying an instrumental variable [21], an observed factor that influences whether an intervention was received, but otherwise does not influence the outcome of interest.

## Supporting Information

S1 Table Results for average careseeking from Health Surveillance Assistants (HSA) and for average careseeking from all sources in Malawi in 2010 and in 2014, in association with the logarithm of the number of HSA in each district. (DOCX)
