# Supplementary material for: Hierarchical Statistical Models to Represent and Visualize Survey Evidence for Program Evaluation: iCCM in Malawi
Source: PLoS One. 2016 Dec 30;11(12):e0168778. doi: 10.1371/journal.pone.0168778 (PMC5201252; doi:10.1371/journal.pone.0168778)
Supplement: S1 Table — (DOCX) [file pone.0168778.s001.docx]

**Annex**

**Hierarchical Statistical Models to Represent and Visualize Survey Evidence for Program Evaluation: iCCM in Malawi**

Table 1: Results for average careseeking from Health Surveillance Assistants (HSA) in Malawi in 2010 (reference) and in 2014, in association with the logarithm of the number of HSA in each district. Conditional on child age, mother’s education, district population of children under five. Models A and C specify the logarithm of district under five population, and models B and D specify a spline of the log of under five population. Models A and B are for all districts in Malawi except Likoma, while Models C and D are for all districts except Likoma and Zomba districts.

|  | **Model A** | | **Model B** | | **Model C** | | **Model D** | |
| --- | --- | --- | --- | --- | --- | --- | --- | --- |
|  | **Est** | **SE** | **Est** | **SE** | **Est** | **SE** | **Est** | **SE** |
| *Fixed Effects* |  |  |  |  |  |  |  |  |
|  |  |  |  |  |  |  |  |  |
| Intercept | -4.64 | 0.81 | -3.68 | 0.90 | -3.05 | 0.99 | -2.07 | 1.08 |
| Log(nHSA) x I(2014) | 0.64 | 0.24 | 0.62 | 0.23 | 0.96 | 0.29 | 0.94 | 0.28 |
| Log(nHSA) | 0.01 | 0.17 | -0.22 | 0.19 | -0.35 | 0.22 | -0.60 | 0.23 |
| I(2014) | -1.50 | 1.13 | -1.39 | 1.05 | -2.87 | 1.34 | -2.77 | 1.26 |
| Log(U5 Population) | -0.15 | 0.16 |  |  | -0.07 | 0.16 |  |  |
|  |  |  |  |  |  |  |  |  |
| Spline of Log(U5 Population) |  |  |  |  |  |  |  |  |
| Term 1 |  |  | 1.03 | 0.39 |  |  | 1.17 | 0.41 |
| Term 2 |  |  | -0.93 | 0.79 |  |  | -0.83 | 0.80 |
| Term 3 |  |  | -1.36 | 0.44 |  |  | -1.19 | 0.46 |
| *Random Effects* |  |  |  |  |  |  |  |  |
|  |  |  |  |  |  |  |  |  |
| EA Variance | 2.13 |  | 2.16 |  | 2.16 |  | 2.18 |  |
| District Variance | 0.00 |  | 0.18 |  | 0.00 |  | 0.00 |  |

Table 2: Results for average careseeking from any health provider in Malawi in 2010 (reference) and in 2014, in association with the logarithm of the number of HSA in each district. Conditional on child age, mother’s education, district population of children under five. Models A and C specify the logarithm of district under five population, and models B and D specify a spline of the log of under five population. Models A and B are for all districts in Malawi except Likoma, while Models C and D are for all districts except Likoma and Zomba districts.

|  | **Model A** | | **Model B** | | **Model C** | | **Model D** | |
| --- | --- | --- | --- | --- | --- | --- | --- | --- |
|  | **Est** | **SE** | **Est** | **SE** | **Est** | **SE** | **Est** | **SE** |
| *Fixed Effects* |  |  |  |  |  |  |  |  |
|  |  |  |  |  |  |  |  |  |
| Intercept | 0.84 | 0.33 | 1.15 | 0.30 | 0.89 | 0.39 | 1.20 | 0.40 |
| Log(nHSA) x I(2014) | 0.02 | 0.08 | 0.02 | 0.09 | 0.07 | 0.09 | 0.08 | 0.09 |
| Log(nHSA) | -0.03 | 0.07 | 0.02 | 0.06 | -0.04 | 0.08 | 0.01 | 0.09 |
| I(2014) | -0.12 | 0.38 | -0.12 | 0.40 | -0.34 | 0.43 | -0.35 | 0.43 |
| Log(U5 Population) | -0.29 | 0.07 |  |  | -0.29 | 0.07 |  |  |
| Spline of Log(U5 Population) |  |  |  |  |  |  |  |  |
| Term 1 |  |  | -0.70 | 0.14 |  |  | -0.69 | 0.18 |
| Term 2 |  |  | -1.03 | 0.28 |  |  | -0.99 | 0.33 |
| Term 3 |  |  | -0.50 | 0.13 |  |  | -0.51 | 0.16 |
| *Random Effects* |  |  |  |  |  |  |  |  |
|  |  |  |  |  |  |  |  |  |
| EA Variance | 0.27 |  | 0.27 |  | 0.26 |  | 0.26 |  |
| District Variance | 0.02 |  | 0.00 |  | 0.02 |  | 0.01 |  |
| District x I(2014) Variance | 0.01 |  | 0.00 |  | 0.01 |  | 0.01 |  |
| Correlation | 0.03 |  | 0.03 |  | 0.03 |  | 0.03 |  |
